# Metabarcoding analysis of trophic sources and linkages in the plankton community of the Kuroshio and neighboring waters

**DOI:** 10.1038/s41598-021-02083-8

**Published:** 2021-12-01

**Authors:** Toru Kobari, Yusuke Tokumo, Ibuki Sato, Gen Kume, Junya Hirai

**Affiliations:** 1 Faculty of Fisheries, Kagoshima University, 4-50-20 Shimoarata, Kagoshima, 8900056 Japan; 2 Graduate School of Agriculture, Forestry and Fisheries, Kagoshima University, 4-50-20 Shimoarata, Kagoshima, 8900056 Japan; 3 Atmosphere and Ocean Research Institute, The University of Tokyo, 5-1-5 Kashiwanoha, Kashiwa, 277-8564 Japan

**Keywords:** Ocean sciences, Marine biology

## Abstract

Trophic sources and pathways supporting early life stages are crucial for survival of forage fishes recruiting around the oligotrophic and unproductive Kuroshio. However, information is limited for the Kuroshio planktonic food web and its trophodynamics because of its high biodiversity. Here, we explore trophic sources and linkages in the Kuroshio plankton community using metabarcoding analysis of gut-content DNA for 22 mesozooplankton groups. The major prey was dinoflagellates and calanoids for omnivorous groups, and calanoids and gelatinous organisms for carnivorous groups. Larvaceans and hydrozoans were the most frequently appeared prey for both omnivores and carnivores, whereas they were minor constituents of the available prey in water samples. Although calanoids overlapped as major prey items for both omnivores and carnivores because they were the most available, contributions from phytoplankton and gelatinous prey differed among taxonomic groups. Further analysis of the metabarcoding data showed that in addition to omnivorous copepods like calanoids, gelatinous groups like larvaceans and hydrozoans were important hubs in the planktonic food web with their multiple trophic linkages to many components. These findings suggest that gelatinous organisms are important as supplementary prey and provide evidence of niche segregation on trophic sources among mesozooplankton groups in the Kuroshio.

## Introduction

The Kuroshio is the western boundary current of the North Pacific Subtropical Gyre^1^. This current flows along the continental slope in the East China Sea and passes through the Tokara Strait before flowing northeast along the Pacific coast of Japan^2^. Because of the low nutrient supply associated with the developed thermocline throughout the year^3^, the Kuroshio has low standing stocks and high biodiversity of phyto- and zooplankton^4,5^. Under such conditions, we might expect poor food availability for planktivorous fish larvae and juveniles in the Kuroshio. However, the Kuroshio in the East China Sea (ECS-Kuroshio) and its neighboring waters have been known as a major nursery ground for larvae of various forage fishes such as Japanese sardine^6^, Japanese jack mackerel^7^, chub mackerel^8^ and common squid^9^. These previous findings give rise to the question of why there is high fishery production, including of these forage fishes, under the oligotrophic conditions and low plankton standing stocks around the Kuroshio (see “the Kuroshio Paradox”^10^).

Microbes predominate pelagic trophodynamics in the oligotrophic North Pacific Subtropical Gyre^11^, which is upstream of the ECS-Kuroshio. The pelagic biome comprises a microbial food web represented by complex trophic linkages^12^. Recent findings demonstrate that heterotrophic bacteria, cyanobacteria and haptophytes are major producers in the ECS-Kuroshio^13,14^. These small protists are consumed by micro- and mesozooplankton communities^15–17^. Mesozooplankton dominated by small calanoid copepods^18,19^ are then major prey items for fish larvae^7,20^. Thereby, mesozooplankton is likely functioning as a hub to integrate various trophic sources and allocate prey for fish larvae in the ECS-Kuroshio. Because of the complicated food web with high biodiversity, however, it is difficult to identify trophic sources and linkages covering the many components at lower trophic levels using contemporary methods such as microscopic examination. Indeed, there is very limited information on the detailed trophic relationships of the planktonic food web in the Kuroshio^19^.

Molecular approaches can achieve high taxonomic resolution, even from fragmented organisms or those lacking morphological characteristics. For instance, the metabarcoding method, which involves amplifying a specific genetic region from bulk DNA samples and recovering taxonomic compositions from massive numbers of sequence reads, has been a powerful tool for gut-content analysis^21,22^. The V9 hyper-variable region in the nuclear gene coding 18S ribosomal RNA (18S rRNA) is a common genetic marker for metabarcoding analysis of eukaryotes^23,24^. There are many registered sequences in public databases for the 18S rRNA gene across eukaryotic taxa (e.g., the SILVA databases^25^), and a universal PCR primer pair is available to amplify the short 18S V9 region across eukaryotes^23^. Because of its high taxonomic coverage and resolution, metabarcoding analysis of the 18S V9 region has been frequently used for gut-content analysis to detect prey items, including organisms undetectable by microscopic analysis^26,27^. While the metabarcoding approach has revealed detailed feeding habits and trophic relationships for some mesozooplankton groups in temperate to subarctic waters^28–30^, no information is available for the highly diverse mesozooplankton communities in subtropical waters. Thus, this molecular approach might provide new insight into overlooked trophic sources of the mesozooplankton community and underestimated trophic functions at lower trophic levels under the poor food availability in the Kuroshio ecosystem.

**Figure 1:**
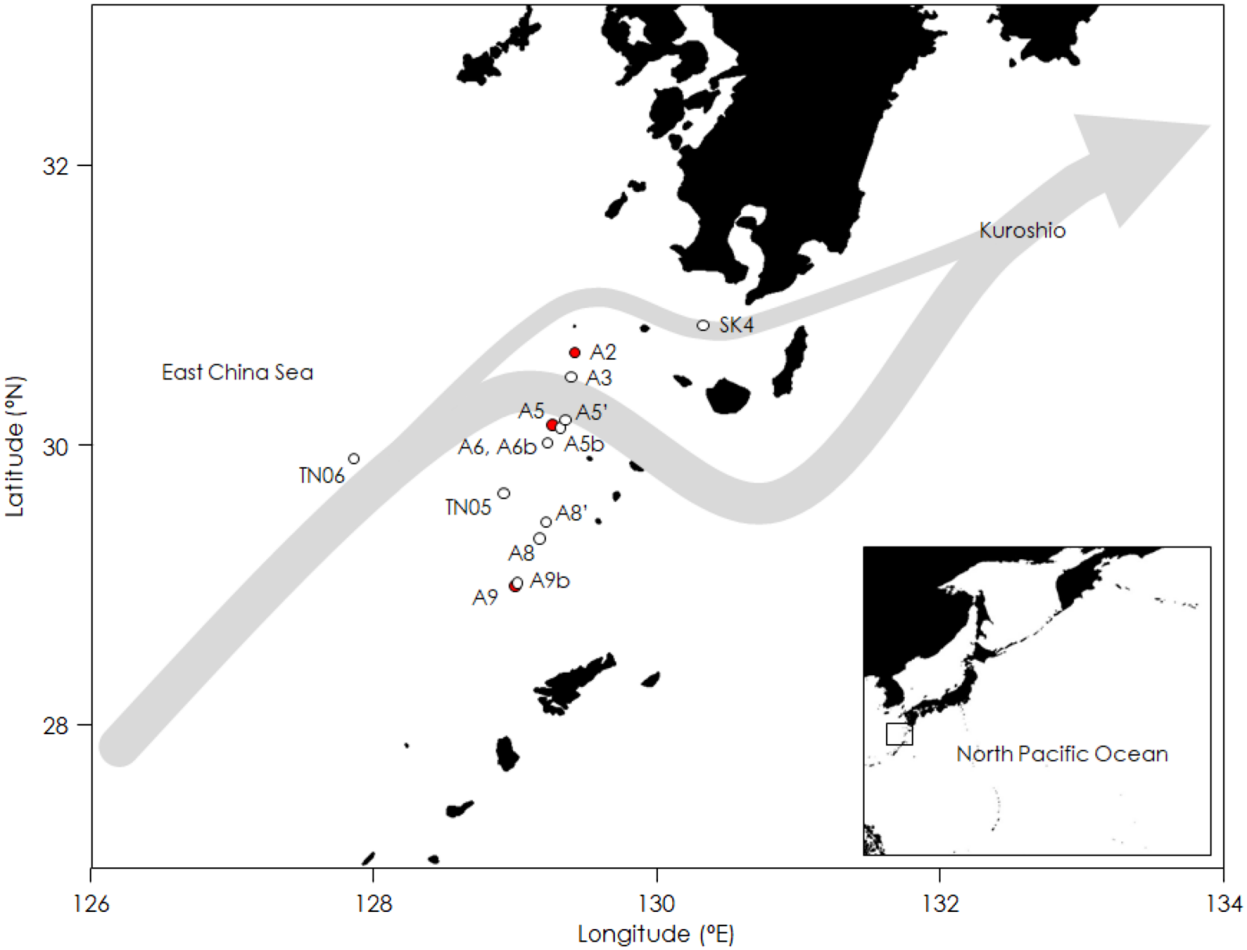
Sampling locations for water samples (solid red circles) and mesozooplankton (solid red and open circles) for metabarcoding analysis in the Kuroshio of the East China Sea and its neighboring waters. Arrows show current direction.

In the present study, we used metabarcoding analysis to identify gut contents of omnivorous and carnivorous mesozooplankton in the ECS-Kuroshio (Fig. 1) to explore their trophic sources under the poor food availability. Water samples were also analyzed to identify the prey available to the mesozooplankton community, and to reveal any prey preferences. Next, we investigated niche segregation on trophic sources for the mesozooplankton community using multivariate analysis of gut-content genes. Finally, we diagrammed the trophic networks among mesozooplankton groups to find major trophic sources and linkages of the planktonic food web in the ECS-Kuroshio.

## Results

### Sequence data

After a quality check of all sequence data, we obtained a total of 6,792,218 reads from gut-content analysis. Ten operational taxonomic units (OTUs) with the most dominant sequence reads (SRs) among the sequence data were classified as major prey. The major prey OTUs comprised 2–60% of all prey OTUs in mesozooplankton gut contents (Supplementary Fig. S1). The remainder were attributed to fungi (~ 27%), craniata (~ 29%) and others including the host and minor prey (24–97%).

### Environmental waters

**Figure 2:**
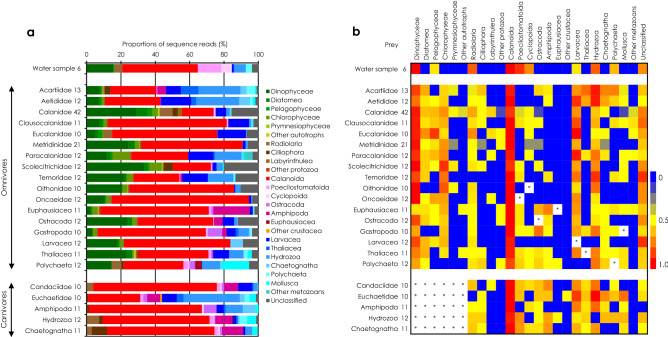
Average proportions for the standardized sequence reads (**a**) and heatmap for average appearance frequency (**b**) of ten dominant prey OTUs from the DNA of ambient water samples (combined) and gut contents of mesozooplankton taxonomic groups in the Kuroshio in the East China Sea and its neighboring waters. Numbers beside group names are the number of replicates. Asterisks indicate eliminated prey groups.

We determined the proportions of SRs for the dominant OTUs in the environmental plankton community (i.e., water samples) (Fig. 2a). The proportions of the available prey in water samples were similar among the stations at 50% Bray–Curtis similarity (Supplementary Fig. S2). Calanoids were the most available prey (~ 56%), and poecilostomatoids (~ 26%) or dinoflagellates (~ 29%) were next. On average, copepods comprised 64% of the available prey, whereas contributions of phytoplankton groups (16%) were similar to those of gelatinous organisms (12%) predominantly hydrozoans. The appearance frequencies of the available prey were compared among water samples (Fig. 2b). Dinoflagellates and calanoids were found in all water samples. Radiolarians, poecilostomatoids, cyclopoids and hydrozoans were next most common, showing high appearance frequencies.

### Higher-taxon-specific prey preferences

The proportions of prey ingested by omnivorous and carnivorous mesozooplankton groups were much different than the proportions in water samples (Fig. 2a). However, calanoids predominated in water samples and were always substantial prey for both omnivorous and carnivorous groups. Except for the Acartiidae and Aetididae, the omnivorous copepod families primary ingested calanoids or dinoflagellates. In any case, phytoplankton and copepods comprised more than half of their ingested prey. Phytoplankton prey was more predominant than calanoids among the ingested prey for Calanidae and Scolecitrichidae. Acartiid and Aetidid copepods exhibited a preference for gelatinous prey, which represented more than half of their ingested prey, with hydrozoans being predominant. High contributions of gelatinous prey were also found for Paracalanidae (33%) and Temoridae (31%). For the other omnivorous groups, calanoids were the predominant ingested prey. Dinoflagellates were the next most common prey for ostracods, larvaceans and thaliaceans. For carnivorous groups, calanoids comprised 62–72% of the ingested prey, except for the Euchaetidae, which ingested similar amounts of hydrozoans and calanids. Protozoans and amphipods accounted for large proportions of the prey ingested by gelatinous carnivores.

For omnivorous mesozooplankton groups, appearance frequencies were high for dinoflagellates and calanoids, and corresponded to the prey availability in water samples (Fig. 2b). The most frequently appearing prey was calanoids for all omnivorous groups. Dinoflagellates were a common prey taxon, with a high appearance frequency for copepods. Besides these frequently appearing prey, chlorophytes and gelatinous prey represented by larvaceans and hydrozoans appeared frequently in the gut contents of omnivorous mesozooplankton groups, whereas chlorophytes were rare in water samples. For carnivorous groups, the most frequently appearing prey was calanoids. The next most frequent prey taxa were larvaceans and hydrozoans, and carnivorous groups ingested other minor prey in water samples, such as amphipods and poecilostomatoids.

### Species-specific preferences

**Figure 3:**
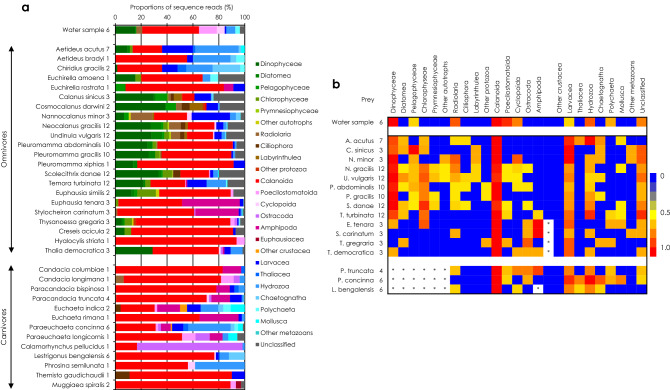
Average proportions of the standardized sequence reads (**a**) and a heatmap of average appearance frequency (**b**) for ten dominant prey OTUs from the DNA of ambient water samples (combined) and gut contents of mesozooplankton species in the Kuroshio in the East China Sea and its neighboring waters. Numbers beside group names are the number of replicates. Asterisks indicate eliminated prey groups.

Prey preference generally differed among congeneric or intergeneric species. The proportions of the dominant prey for omnivorous and carnivorous mesozooplankton species were much different than the proportions in water samples (Fig. 3a). As in comparisons among higher taxonomic groups, calanoids predominated in water samples and often contributed substantially to the ingested prey for both omnivorous and carnivorous species. Besides the predominant prey, there were species-specific proportions of additional ingested prey items for omnivorous and carnivorous species. For example, *Aetidius*, *Chiridius*, *Nannocalanus* and *Temora* species were characterized by high contributions of gelatinous prey. Calanidae species exhibited wide prey spectra and different preferences for secondary prey, such as dinoflagellates, protozoans and larvaceans. For the omnivores other than copepods, the contributions of amphipods or dinoflagellates provided species-specific differences in prey proportions. For carnivorous species, their specific prey proportions resulted from the contributions of crustacean and gelatinous prey. For example, among carnivorous copepods, amphipods or hydrozoans were the next most contributed prey and their contributions to the ingested prey were different. Similarly, the next most predominant frequent prey differed among amphipod species.

Dinoflagellates and calanoids had the highest appearance frequencies among the ingested prey of omnivorous species, and calanoids had the highest appearance frequencies among the ingested prey of carnivorous species, corresponding to their appearance frequencies in water samples (Fig. 3b). In addition to dinoflagellates and calanoids, chlorophytes appeared frequently among the ingested prey of omnivorous species, and gelatinous organisms (represented by larvaceans and hydrozoans) appeared frequently among the prey of both omnivorous and carnivorous species.

### Multivariate analysis of gut contents

**Figure 4:**
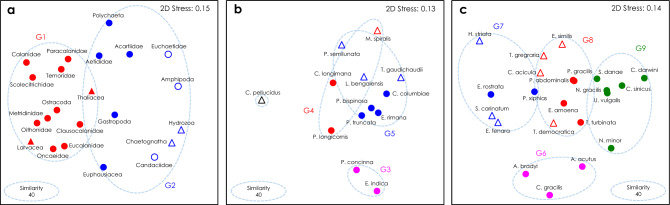
Non-metric multi-dimensional scaling (NMDS) ordination plot of the standardized sequence reads of ten dominant prey OTUs in gut-content DNA of mesozooplankton groups (**a**), carnivorous species (**b**) and omnivorous species (**c**) based on Bray–Curtis similarity. Groups within broken lines (G1 to G9) show clusters classified on the bases of similarity of prey proportions. Symbols with the same colors belong in same cluster. (**a**) Omnivorous (solid symbols) and carnivorous (open symbols) groups. (**b**,**c)** Calanoids (circles) and other (triangles) groups.

**Figure 5:**
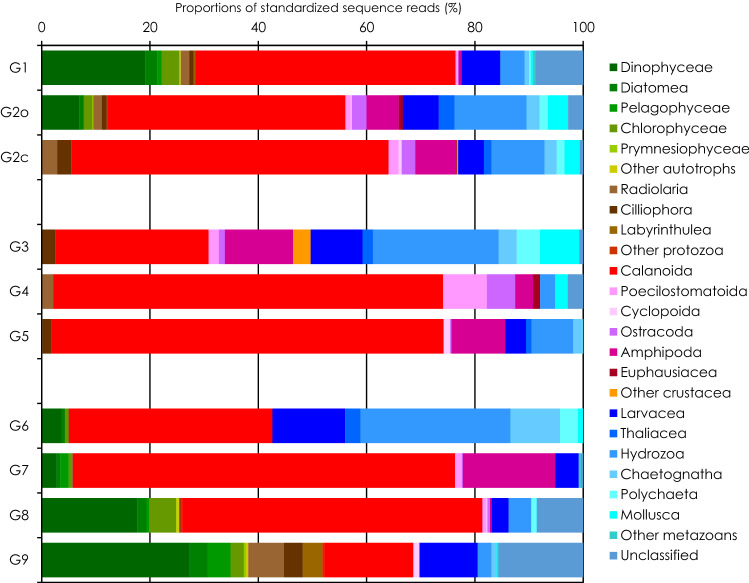
Average proportions of the standardized sequence reads for ten dominant prey OTUs presented for nine groups (G1 to G9) classified by Bray–Curtis similarity on a non-metric multi-dimensional scaling ordination plot. G2o: omnivorous groups for G2. G2c: carnivorous groups for G2.

We visualized the multivariate occurrence patterns of gut-content DNA among higher taxonomic groups and species using a non-metric multi-dimensional scaling (NMDS) plot on the standardized SRs of the major prey OTUs (Fig. 4). There was little overlap between omnivorous and carnivorous groups. Carnivorous groups were grouped to the right side of the plot due to their preferences for crustacean prey. Omnivorous calanoids were allocated toward the left side of the plot and segregated from the carnivores because of their preferences for phytoplankton prey. Omnivorous copepods (i.e., Aetididae and Acartiidae) and polychaetes exhibiting a preference for gelatinous prey, were found at the top of the plot between the carnivorous and omnivorous groups. The mesozooplankton community was classified into two groups (G1 and G2) based on 40% Bray–Curtis similarity for prey proportions of the gut-content DNA. Besides the predominant prey (i.e., calanoids), their prey proportions were distinguished by the next most contributed prey, which were dinoflagellates for G1 and hydrozoans for G2 (Fig. 5).

We next prepared NMDS plots for the standardized SRs of the ingested prey among carnivorous and omnivorous mesozooplankton species (Fig. 4b,c). The carnivorous species were widely scattered across the plot. Carnivorous calanoid species were allocated toward the lower side of the plot, whereas the other carnivorous species were closer to the upper side (Fig. 4b). As 40% Bray–Curtis similarity for prey proportions, carnivorous species were classified into three groups (G4, G5 and G6), except for outliers. Besides calanoids, with the highest contributions, their prey proportions were characterized by a higher contribution from gelatinous prey for G3 and from crustacean prey for G4, than for G5 (Fig. 5). Most of the omnivorous species were scattered around the NMDS plot, with overlapping for some Calanidae (*Neocalanus gracilis* and *Undinula vulgaris*) and Metridinidae species (*Pleuromamma gracilis* and *P. abdominalis*) (Fig. 4c). Omnivorous calanoid species were plotted toward the right side, while *Aetidius*　and *Chiridius* species were found at the bottom and segregated from the other omnivorous copepods because of their preference for gelatinous prey. The other omnivorous species were allocated toward the left side because their prey spectra were narrower than those of copepods. At 30% Bray–Curtis similarity, the omnivorous species were classified into four groups (G6, G7, G8 and G9). Their prey proportions were characterized by the next most contributed prey after the highest contribution from calanoids: gelatinous prey represented by larvaceans and hydrozoans for G6, amphipods for G7, and dinoflagellates for G8. G9 had the highest contribution from dinoflagellates and wider prey spectra than the other groups.

### Trophic linkages of the plankton community

**Figure 6:**
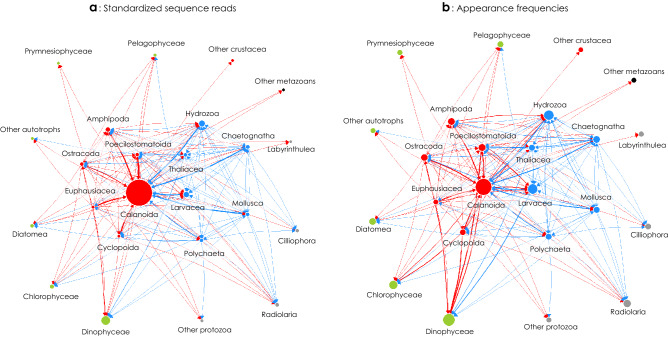
Trophic linkages of the plankton community in the Kuroshio of the East China Sea and its neighboring waters based on the standardized sequence reads (**a**) and appearance frequencies (**b**) of ten dominant prey OTUs in gut contents of omnivorous and carnivorous mesozooplankton groups. Arrow indicates the direction of energy flow from predator to prey. The thickness of each arrow reflects the number of relationships between prey and predator and the size of each circle corresponds to the relative importance of the trophic relationships among the other components determined with the Page Rank algorithm (https://igraph.org/r/doc/page_rank.html) from the R. Red for crustaceans, blue for gelatinous forms, green for autotrophs, gray for protozoans, black for other metazoans.

Using the standardized SRs and appearance frequencies of the major prey, we constructed networks of trophic linkages (i.e., prey–predator relationships) for mesozooplankton groups and their prey components. Trophic interactions differed slightly between the network based on standardized SRs and the network based on the appearance frequencies. For the standardized SRs (Fig. 6a), calanoids were an important node having the most trophic linkages with the various mesozooplankton groups as both predator and prey, including the other calanoid families. They had no specific trophic linkage to the other prey groups because of their wide prey spectra. The next most important nodes in terms of the number of trophic linkages were larvaceans and hydrozoans. In the network based on appearance frequencies, calanoids were still an important node because they were the most frequent prey for the various mesozooplankton groups, but the next most important nodes were dinoflagellates, larvaceans, and hydrozoans (Fig. 6b). Compared to the network based on the standardized SRs, dinoflagellates, larvaceans and hydrozoans in the network based on appearance frequencies had a higher number of trophic linkages to other constituents because of their frequent appearances as supplementary prey.

## Discussion

Using a DNA-based approach, we have characterized the diets of 22 higher taxonomic groups, 13 copepod families and 35 species, that dominate in the Kuroshio food web. This technique was much more efficient than traditional analysis for detecting the various prey items of the mesozooplankton community across wide taxonomic groups; microscopic analysis of gut contents requires extensive time and effort and much higher skills for taxonomic identification. In particular, this DNA-based approach was effective for identifying fragile and fragmented prey, which are otherwise difficult or impossible to classify in mesozooplankton gut contents. We confirmed that the major prey was calanoid copepods for both omnivores and carnivores. Our findings also highlight that dinoflagellates and gelatinous groups are ingested by various mesozooplankton groups as supplementary prey.

Multivariate analysis confirmed that there is overlap in the major preys, but the supplementary preys suggest niche segregation on trophic sources among carnivorous and omnivorous mesozooplankton groups. On the basis of these findings, this study is to identify in detail the trophic network of the planktonic food web in the Kuroshio. This DNA-based approach was quantitative, using standardized indices to demonstrate trophic linkages among the diverse taxonomic groups. Notably, this approach emphasized the importance of gelatinous organisms, which have long been overlooked and/or underestimated as both prey and predator in the planktonic food web. We highlighted the major trophic hubs of the planktonic food web in the Kuroshio, specifically calanoid copepods and gelatinous organisms represented by larvaceans and hydrozoans, because of their multiple trophic linkages.

From a methodological point of view, some attention should be paid to the relative contributions of SRs among prey items in our results. First, our method would underestimate pico-sized eukaryotes not retained on the filters used. However, these small eukaryotes are not predominant components of phytoplankton community^14^ and likely minor prey for mesozooplankton in the Kuroshio^15^. A second issue might be variable copy numbers of 18S rRNA genes among taxonomic groups of eukaryotic plankton. For example, dinoflagellates possess a large number of copies of the 18S rRNA genes compared with other phytoplankton groups because of their phyletic evolution with symbiosis^24^. This means that the relative contributions of dinoflagellate genes to gut-content genes of mesozooplankton might be overestimated, compared with the genes of other phytoplankton. Indeed, high contributions of dinoflagellates have also been found in the gut-content genes of copepods^28^ and pelagic tunicates^31^, and both of these studies noted some overestimates for dinoflagellates. In contrast, larvaceans have a smaller genome compared with other mesozooplankton groups^32^, indicating the possibility of some underestimation. These characteristics probably resulted in the lower apparent contributions but more frequent appearances of larvaceans in gut-content genes of ichtyoplankton^33^. There are currently no corrections or calibrations for these variations in 18S rRNA genes among taxonomic groups, and thus the relative contributions might be overestimated for dinoflagellates and underestimated for larvaceans. There might also be biases in our relative prey contributions resulting from PCR, because we did not use PCR replicates. Although PCR biases can be problematic, especially in detecting rare OTUs^34,35^, more reliable results for major mesozooplankton prey could be obtained by using replicate samples. In addition, there is a potential risk of contamination in metabarcoding analysis, such as the inclusion of non-food DNA, laboratory contamination, symbionts of prey, and secondary predation^36^. For example, phytoplankton OTUs in carnivorous chaetognath guts, which were assumed to be contaminations, were present with variable read abundance (~ 14%; Supplementary Fig. S2). While such biases and contaminants should not be ignored, these phytoplankton OTUs averaged less than 3% of the total SRs, which was lower than the proportions of major prey OTUs. In this study, we focused on major OTUs and analyzed not only read abundance but also appearance frequency to avoid problems from these contaminants. However, future studies might obtain more reliable metabarcoding data by using control samples to evaluate the influence of contamination.

Phytoplankton prey made higher contributions to the gut contents of ostracods, thaliaceans and some calanoids (Calanidae, Metridinidae and Scolecitrichidae), compared to their relatively minor proportions among available prey in the Kuroshio. This suggests a more herbivorous feeding habit among these omnivorous mesozooplankton groups. On the other hand, the proportions of calanoids in the ingested prey of most omnivorous groups were higher than those in ambient water. Since mesozooplankton feeding in the Kuroshio show no specific prey preference for copepod nauplii^15^, these relatively high contributions are unlikely to reflect carnivorous feeding on calanoids. Rather, considering the high proportions of calanoids as prey in gut contents of Oithonidae, which are known to be coprophagous^37–39^, these omnivorous groups might be feeding on fecal pellets egested from calanoid copepods. Indeed, fecal pellets egested from calanoids are known to be consumed by these small copepods in neighboring waters^40^. Support for coprophagy on calanoid fecal pellets might be evident in the similar contributions of calanoids in water samples and gut contents of larvaceans, known as opportunistic filter feeders.

One notable result from the present study is that gelatinous organisms appeared in the gut contents not only of carnivorous but also of omnivorous mesozooplankton. Larvaceans and hydrozoans appeared frequently among the ingested prey. Such frequent appearances of gelatinous organisms have been reported for omnivorous copepods: *Calanus sinicus* around the Kuroshio^41^ and *C. finmarchicus* in the North Atlantic Ocean^28^. As gelatinous organisms were found in our water samples, they might be ingested by omnivores in the form of particles, such as fragmented bodies, discarded houses and egested fecal pellets. Because of the difficulty of identifying prey from microscopic analysis of these fragile particles with no morphological characteristics, these gelatinous organisms might have been previously overlooked as mesozooplankton prey. Observations of feeding behavior show that gelatinous organisms are prey for poecilostomatoid copepods^42^ and amphipods^43^. Moreover, discarded larvacean houses are major prey items for poecilostomatoid copepods^44^. Because larvaceans exhibit high production rates of houses despite their small standing stocks^45^, these discarded houses would be widely available prey for omnivorous mesozooplankton, as reflected in their appearance frequencies. Given the difficulty of their morphological identification in gut contents, the importance of gelatinous taxa as prey for both omnivorous and carnivorous mesozooplankton in the Kuroshio should be highlighted more.

Both calanoids and gelatinous organisms are abundant in the mesozooplankton community in the Kuroshio^18^. However, calanoid fecal pellets and gelatinous prey have not been identified as trophic sources for omnivorous copepods because of the difficulty of identifying them in gut contents. Based on the carbon budget for the planktonic food web in the Kuroshio^19^, autotrophic and heterotrophic protists cannot support the carbon requirements of the mesozooplankton community. We suggest that calanoid fecal pellets and gelatinous prey are overlooked food sources helping to support the mesozooplankton community in the Kuroshio.

The prey proportions of both omnivorous and carnivorous mesozooplankton groups and species were often different from that of the ambient prey in water samples, indicating specific prey preferences. Whereas the predominant prey overlapped among omnivorous and carnivorous groups, we found higher-taxon-specific or species-specific preferences for their supplementary prey. From this, trophic sources could be segregated on the basis of their supplementary prey, such as phytoplankton, amphipods or gelatinous prey. For example, despite the similar contributions from the predominant prey (i.e., calanoids), the prey proportions of the secondary prey permitted segregation, such as phytoplankton and gelatinous prey for omnivores, and amphipods and gelatinous prey for carnivores. On the NMDS scatter plots for the higher taxonomic groups or omnivorous groups, Acartiid and Aetidid copepods were clearly segregated from the other omnivorous groups with stronger preferences for gelatinous prey. Such prey-based niche segregation was also evident in species-level comparisons. Carnivorous species were widely scattered on the NMDS plot even among congeneric species because of their species-specific preferences for supplementary prey as groups (i.e., crustaceans or gelatinous forms) and in combination. Omnivorous calanoid species were also scattered because of variable species-specific preferences for phytoplankton and gelatinous prey. These findings suggest that niche segregation on trophic source is facilitated by a variety of supplementary prey.

Previous studies have shown low phytoplankton standing stocks and a predominance of pico- to nano-autotrophs in the Kuroshio^13,14,19^, suggesting that phytoplankton prey might not be sufficient to support the mesozooplankton community. Nevertheless, dinoflagellates were frequently ingested as prey by most omnivorous mesozooplankton groups and species. Although phytoplankton standing stocks and productivity in the Kuroshio are stimulated by a supply of nutrients^16,46,47^ and intermittently increase through advection from coastal communities^19^, mesozooplankton feeding experiments show no significant ingestion of autotrophic dinoflagellates^15^. Estimates of the carbon budget have demonstrated that heterotrophic nanoflagellates contributed to mesozooplankton food requirements^19^. If the dinoflagellates found in the gut contents of omnivorous groups are assumed to be nano-sized heterotrophs, then their presence as frequently ingested prey in this study is reasonable because heterotrophic nanoflagellates can contribute to the carbon requirements of the mesozooplankton community in the Kuroshio^19^. On the other hand, previous experiments have demonstrated that microzooplankton consume half of phytoplankton production and account for the major pathways of the planktonic food web in the ECS-Kuroshio^17^. Considering the preference for naked ciliates in mesozooplankton feeding experiments^15^, the path from ciliates to omnivorous copepods is expected to be one of the major trophic pathways in the Kuroshio. Contrary to such expectation, our DNA-based approach shows this trophic pathway is likely to be relatively minor, because the standardized SRs and the appearance frequencies of ciliates in mesozooplankton gut contents were low. Similarly, ciliates were minor components of the gut contents of *C. sinicus* and the environmental plankton community in the neighboring waters of the Kuroshio^41^. It is possible that naked ciliates in the Kuroshio were underestimated because of lower detection with metabarcoding analysis^24^ and their fragile cells^48^. However, a previous study showed that ciliates had low standing stocks in the plankton community and could not support mesozooplankton respiratory requirements^19^. The trophic linkages of the plankton community derived from our study show that the trophodynamics from ciliates to omnivorous copepods might be relatively minor for the Kuroshio planktonic food web.

Specific preferences for pelagic tunicates as prey have been reported for Sapphirinidae copepods (poecilostomatoids), which feed on doliolids in the Kuroshio Extension^42^, and Oncaeidae copepods (poecilostomatoids), which feed on discarded larvacean houses in the Kuroshio^44^. These studies have pointed out that the prey preferences for pelagic tunicates are important for biogeochemical cycling in the Kuroshio. Pelagic tunicates like doliolids and larvaceans efficiently ingest small particles like cyanobacteria and heterotrophic bacteria because of their fine feeding filters^49,50^, and these microbes have high standing stocks in the Kuroshio^19^. Thus, pelagic tunicates have come under focus as secondary producers for integrating microbial production in the Kuroshio^10^. Their further trophic network connections to various mesozooplankton groups are supported by our DNA-based approach. On the other hand, hydrozoans exhibited interactive trophic relationships with copepods as both predator and prey. Recognizing that hydrozoans are one of the most frequent prey for many mesozooplankton groups, the trophic networks of the planktonic food web in the Kuroshio are more complicated than suggested by previous studies^19^ because of the interactive relationships among many taxonomic groups. We suggest that gelatinous organisms are an important trophodynamic hub that characterize the planktonic food web in the Kuroshio.

## Conclusions

Our results demonstrate that the major prey items in the Kuroshio planktonic food web are calanoid fecal pellets for omnivorous mesozooplankton and crustaceans for carnivores. Dinoflagellates, larvaceans and hydrozoans are also ingested as supplementary prey for omnivores and carnivores. However, the contribution of these supplementary prey relative to the major prey taxa varies by taxonomic group. These findings based on metabarcoding datasets highlight niche segregation on trophic sources among various taxonomic groups in the Kuroshio. Further analysis of these metabarcoding datasets shows that omnivorous calanoids, larvaceans and hydrozoans are important hubs of the planktonic food web because of their trophic linkages with multiple components.

To date, there has been limited information on trophic sources and linkages in the planktonic food web in the Kuroshio ecosystem because contemporary methodologies require considerable effort, time, and taxonomic-identification skills for application to the highly diverse Kuroshio ecosystem. However, a DNA-based approach is extremely efficient and effective for covering a wide range of taxonomic groups in the plankton community. Moreover, these metabarcoding datasets provide new insights for exploring the groups of fragile or fragmented organisms that have been previously overlooked, for evaluating niche segregation of mesozooplankton based on trophic sources, and for visualizing the trophic network of the planktonic food web. Such approach can provide further understanding of the Kuroshio ecosystem of which much is still unknown.

## Methods

### Sample collection

We collected mesozooplankton samples for gut content analysis at nine stations and water samples to determine ambient prey at three different stations in the ECS-Kuroshio during cruises aboard the training ship (T/S) *Kagoshima Maru* and T/S *Nansei Maru* from 2015 to 2019 (Fig. 1). Mesozooplankton were collected vertically in the layer shallower than 200 m with a single or twin-type North Pacific Standard Net (mesh opening, 0.1 mm; mouth diameter, 450 mm). These mesozooplankton samples were quickly frozen and stored at − 20 °C. Water samples were collected from the subsurface chlorophyll maximum to investigate prey availability for mesozooplankton using X-Niskin bottles attached to a CTD sampling system (SBE-911 Plus, Sea-Bird Electronics). The collected seawater (1000 mL) was immediately filtered through a hydrophilic PTFE membrane filter (5 µm, Merck). The filters were stored at − 80 °C for later analysis.

### High-throughput sequencing

For metabarcoding, we used 292 individuals from 22 mesozooplankton groups, including 13 copepod families for gut-content analysis (Table 1), and six filters from water samples for ambient prey of mesozooplankton. These mesozooplankton groups, and copepods in particular, are predominant components of the mesozooplankton community^18,19^, and are prey items for fish larvae in the Kuroshio and its neighboring waters^7,20^. These individuals were widely collected from stations ranging from the continental slope waters to offshore of the Kuroshio path (Supplementary Table S1). Based on 50% Bray–Curtis similarity, the proportions of available prey were similar among the stations and predominated by dinoflagellates, calanoids, and poecilostomatoids (Supplementary Fig. S2), due to the similar environments in the study area (Supplementary Table S2). All filter samples were thus treated as the “water sample”, although we collected these samples at different stations.

**Table 1:** Mesozooplankton taxonomic groups identified in gut contents in the present study. Taxonomic groups eliminated as host or contaminated organisms are also indicated.

Taxon	Feeding habit	Species, genus or groups	Eliminated taxonomic groups
Chaetognatha	Carnivore	Sagittidae spp.	Chaetognatha, Phytoplankton, Craniata, Fungi
**Copepoda**			
***Calanoida***			
Acartiidae	Omnivore	*Acartia* spp.	Acartiidae, Craniata, Fungi
Aetididae	Omnivore	*Aetideus acutus, A. bradyi, Chiridius gracilis, Euchirella amoena, E. rostrata*	Aetididae, Craniata, Fungi
Calanidae	Omnivore	*Calanus sinicus, Cosmocalanus darwini, Nannocalanus minor, Neocalanus gracilis, Neocalanus* spp.*, Undinula vulgaris*	Calanidae, Craniata, Fungi
Candaciidae	Carnivore	*Candacia columiae, C. longimana, Paracandacia bispinosa, P. truncata,* Candaciidae spp.	Candaciidae, Phytoplankton, Craniata, Fungi
Clausocalanidae	Omnivore	*Clanusocalanus* spp.	Clausocalanidae, Craniata, Fungi
Eucalanidae	Omnivore	*Eucalanus* spp.	Eucalanidae, Craniata, Fungi
Euchaetidae	Carnivore	*Euchaeta indica, E. rimana, Paraeuchaeta concinna, P. longicornis*	Euchaetidae, Phytoplankton, Craniata, Fungi
Metridinidae	Omnivore	*Pleuromamma xiphias, P. gracilis, P. abdominalis*	Metridinidae, Craniata, Fungi
Paracalanidae	Omnivore	*Paracalanus* spp.	Paracalanidae, Craniata, Fungi
Scolecitrichidae	Omnivore	*Scolecithrix danae*	Scolecitrichidae, Craniata, Fungi
Temoridae	Omnivore	*Temora turbinata*	Temoridae, Craniata, Fungi
***Cyclopoida***			
Oithonidae	Omnivore	Oithonidae spp.	Oithonidae, Craniata, Fungi
**Poecilostomatoida**			
Oncaeidae	Omnivore	*Oncaea* spp.	Oncaeidae, Craniata, Fungi
Gastropoda	Omnivore	*Creseis acicula, Hyalocylis striata*, Gastropoda spp.	Gustropoda, Craniata, Fungi
Hydrozoa	Carnivore	*Muggiaea spiralis*, Hydrozoa spp.	Hydrozoa, Phytoplankton, Craniata, Fungi
Larvacea	Omnivore	Oikopleura spp.	Larvacea, Craniata, Fungi
**Malacostraca**			
Amphipoda	Carnivore	*Calamorhynchus pellucidus, Lestrigonus bengalensis, Phrosina semilunata, Themisto gaudichaudii,* Hypediidae spp.	Amphipoda, Phytoplankton, Craniata, Fungi
Euphausiacea	Omnivore	*Euphausia similis, E. tenara, Stylocheiron carinatum, Thysanoessa gregraria*	Euphausiasea, Craniata, Fungi
Ostracoda	Omnivore	*Conchoecia* spp.	Ostracoda, Craniata, Fungi
Polychaeta	Omnivore	Polychaeta spp.	Polychaeta, Craniata, Fungi
Thaliacea	Omnivore	*Thalia democratica*, Salpidae spp.	Thaliacea, Craniata, Fungi

After the frozen mesozooplankton samples were thawed, the target specimens were sorted from the bulk samples under a dissecting microscope (SMZ1800, Nikon) and washed with filtered seawater (0.2-μm pore-size cartridge filter, Advantec). The whole gut was removed from each individual to avoid loss of gut contents. Genomic DNA was extracted in a 1.5-mL tube containing 30 μL (gut contents) or 200 μL (water samples) of 5% Chelex buffer (Bio-Rad). These samples were homogenized with a pellet pestle and heated at 95 °C for 20 min. The samples were then centrifuged at 10,000 × *g* for 1 min. DNA concentrations in the supernatants were measured using a Qubit Assay Kit (Thermo-Fisher Scientific) and a library was prepared using high-throughput sequencing based on the three-step PCR method^21^. The 18S rRNA V9 region was amplified using eukaryotic universal primers 1389F and 1510R^23^, and adaptor and dual-index sequences were attached during second and third PCRs for sequencing runs on an Illumina MiSeq. Each PCR sample was prepared in a 15 μL reaction volume using KOD Plus version 2 (Toyobo Inc.) (Supplementary Table S3). PCR conditions followed a previous method^21^ (Supplementary Table S4). PCR products from the target region were confirmed by electrophoresis using a 2.0% agarose gel after first and third PCRs. Final PCR products were purified with a QIAquick PCR Purification Kit (Qiagen), and the concentrations of purified PCR products were measured with a Qubit Assay Kit. The quality of final PCR products was confirmed by using a Bioanalyzer (Agilent), and high throughput sequencing runs were performed using the MiSeq Reagent Kit v2 (Illumina) on an Illumina MiSeq to obtain 2 × 250 bp paired-end SRs.

### Bioinformatic analysis

Raw SRs were quality-filtered using Trimmomatic^51^ and paired-end sequences were merged and further quality-filtered in mothur version 1.39.1^52^. After sequence alignment against the SILVA 132 database^25^, single-linkage pre-clustering^53^, and chimera removal using UCHIME^54^ in mothur, taxonomic classifications were performed based on PR2 version 4.14.0^55^ using a naïve Bayesian classifier^56^ with a threshold greater than 70%. As this study focused on eukaryotic organisms, only sequences classified as “Eukaryota” were selected. The taxonomic groups “Craniata” was removed from SRs of gut-content data to avoid contamination. The final quality-filtered sequences were clustered into operational taxonomic units (OTUs). We used the 99% similarity threshold for high taxonomic resolution based on the average neighbor algorithm. Taxonomic resolutions of prey OTUs was determined at a family or higher level. Some taxonomic groups were removed from SRs of gut-content data as reflecting the host or contamination from other organisms (Table 1). To avoid erroneous inflation of minor OTUs and to cover the variety of major prey, we investigated ten dominant OTUs for each sample of mesozooplankton prey or water. Unclassified OTUs in the ten dominant OTUs were verified with the NCBI “nt” database in BLAST (https://blast.ncbi.nlm.nih.gov/Blast.cgi). They were classified to the order level with greater than 99% identity or kept as unclassified prey at 99% or lower identity.

### Data analysis

The proportion and frequency of appearance of SRs for the major prey OTUs were evaluated for each mesozooplankton taxonomic group and for water samples. For computing the proportion of SRs for the major prey OTUs among the different taxonomic groups or species, the number of SRs for each prey OTU for individual mesozooplankton and for each water sample were standardized against the total number of SRs of prey OTUs (i.e., standardized SRs) to correct for the different success of amplifications among the samples. We evaluated niche segregation on trophic sources among mesozooplankton taxonomic groups with multivariate analysis using Primer 6 (Primer-E Ltd.). After square-root transformation of the standardized SRs of major prey OTUs, we constructed a similarity matrix between all mesozooplankton individuals using Bray–Curtis similarity. From this matrix, multivariate patterns were visualized with a non-metric multi-dimensional scaling (NMDS) plot. The trophic networks for mesozooplankton and prey groups were determined with the Page Rank algorithm (https://igraph.org/r/doc/page_rank.html) for R 4.0.0 (http://www.r-project.org/index.html) based on the standardized SRs and appearance frequencies of the major prey OTUs in gut contents of omnivorous and carnivorous mesozooplankton.

## Supplementary Information

Supplementary Information.

## Data Availability

Raw Illumina MiSeq data are available in the NCBI/ EBI/DDBJ Sequence Read Archive (BioProject accession PRJDB11622), which includes the SRA/ERA/DRA accession number for each sample (DRX293498–DRX293477). All data sets and scripts (mothur, Page Rank algorithm) used in this paper are available at figshare (https://doi.org/10.6084/m9.figshare.16802095.v1).
