# Supplementary material for: Metabarcoding analysis of trophic sources and linkages in the plankton community of the Kuroshio and neighboring waters
Source: Sci Rep. 2021 Dec 1;11:23265. doi: 10.1038/s41598-021-02083-8 (PMC8636560; doi:10.1038/s41598-021-02083-8)
Supplement: Supplementary file 1 — Supplementary Information. [file 41598_2021_2083_MOESM1_ESM.docx]

Supplementary Table S1. Sampling locations for mesozooplankton and water samples. Cruise ID stands for abbreviation of the ship, year and month. KG: Kagoshima Maru. NS: Nansei Maru. CSW: Continental slope water. KR: Kuroshio path. OKR: Offshore of Kuroshio path. -: No data.

| Target samples |  |  | CruiseID | | |  | Replicates | | |
| --- | --- | --- | --- | --- | --- | --- | --- | --- | --- |
|  |  |  | CSW | KR | OKR |  | CSW | KR | OKR |
| Water sample |  |  | KG1611 | KG1611 | KG1611 |  | 2 | 2 | 2 |
| Copepoda | Acartiidae | *Acartia* spp. | - | KG1711 | - |  | - | 13 | - |
|  | Aetididae | *Aetideus acutus* | KG1511 | KG1511 | - |  | 2 | 5 | - |
|  |  | *Aetideus bradyi* |  | KG1711 | - |  | - | 1 | - |
|  |  | *Chiridius gracilis* | KG1511 | KG1711 | - |  | 1 | 1 | - |
|  |  | *Euchirella amoena* | - | - | KG1711 |  | - | - | 1 |
|  |  | *Euchirella rostrata* | - | KG1611 | - |  | - | 1 | - |
|  | Calanidae | *Calanus sinicus* | KG1611 | KG1611 | - |  | 1 | 2 | - |
|  |  | *Cosmocalanus* spp. | KG1711 | - | KG1711 |  | 3 | - | 4 |
|  |  | *Cosmocalanus darwini* | - | KG1611 | - |  | - | 3 | - |
|  |  | *Nannocalanus minor* | - | KG1611 | KG1611 |  | - | 2 | 1 |
|  |  | *Neocalanus* spp. | KG1711 | - | - |  | 3 | - | - |
|  |  | *Neocalanus gracilis* | - | KG1711 | KG1611, KG1711 |  | - | 6 | 6 |
|  |  | *Undinula vulgaris* | KG1611, KG1711 | KG1711 | KG1611 |  | 4 | 7 | 1 |
|  | Candaciidae | Candaciidae spp. | KG1711 | - | - |  | 3 | - | - |
|  |  | *Candacia comunbiae* | KG1611 | - | - |  | 1 | - | - |
|  |  | *Candacia longimana* | - | - | KG1611 |  | - | - | 1 |
|  |  | *Paracandacia bispinosa* | - | - | KG1611 |  | - | - | 1 |
|  |  | *Paracandacia truncata* | - | KG1611 | KG1611 |  | - | 3 | 1 |
|  | Clausocalanidae | *Clausocalanus* spp. | NS1706, KG1711 | KG1611 | KG1611 |  | 6 | 2 | 3 |
|  | Eucalanidae | *Eucalanus* spp. | KG1611 | KG1611 | KG1611 |  | 3 | 3 | 3 |
|  |  | *Eucalanus mucronatus* | KG1711 | - | - |  | 1 | - | - |
|  | Euchaetidae | *Euchaeta indica* | KG1711 | - | KG1611 |  | 1 | - | 1 |
|  |  | *Euchaeta rimana* | - | - | KG1611 |  | - | - | 1 |
|  |  | *Paraeuchaeta concinna* | KG1711 | KG1611 | - |  | 3 | 3 | - |
|  |  | *Paraeuchaeta longicornis* | - | - | KG1611 |  |  |  | 1 |
|  | Metridinidae | *Pleuromamma abdominalis* | KG1611 | KG1611, KG1711 | KG1711 |  | 1 | 5 | 4 |
|  |  | *Pleuromamma gracilis* | KG1711 | KG1711 | KG1711 |  | 3 | 3 | 4 |
|  |  | *Pleuromamma xiphias* | KG1611 | - | - |  | 1 | - | - |
|  | Paracalanidae | *Paracalanus* spp. | NS1706, KG1611, KG1711 | KG1611 | - |  | 10 | 2 | - |
|  | Scolecitrichidae | Scolecithrix danae | NS1706, KG1711 | KG1711 | KG1711 |  | 8 | 5 | 1 |
|  | Temoridae | Temora turbinata | NS1706 | KG1711 | - |  | 9 | 3 | - |
|  | Oithonidae | *Oithona* spp. | - | KG1711 | KG1711 |  | - | 6 | 4 |
|  | Oncaeidae | *Oncaea* spp. | KG1611 | KG1711 | KG1611 |  | 3 | 6 | 3 |
| Ostracoda |  | *Conchoecia* spp. | NS1704, NS1706, KG1711 | KG1711 | KG1611 |  | 6 | 3 | 3 |
| Euphausiacea |  | *Euphausia similis* | - | KG1711 | - |  | - | 2 | - |
|  |  | *Euphausia tenara* | KG1611 | - | - |  | 3 | - | - |
|  |  | *Stylocheiron carinatum* | - | - | KG1711 |  | - | - | 3 |
|  |  | *Thysanoessa gregaria* | - | KG1711 | - |  | - | 3 | - |
| Amphipoda |  | Hyperiidae spp. | KG1711 | - | - |  | 2 | - | - |
|  |  | *Calamorhynchus pellucidus* | - | KG1611 | - |  | - | 1 | - |
|  |  | *Lestrigonus bengalensis* | KG1611 |  | KG1711 |  | 3 | - | 3 |
|  |  | *Phrosina semilunata* | - | KG1711 | - |  | - | 1 | - |
|  |  | *Themisto gaudichaudii* | - | KG1611 | - |  | - | 1 | - |
| Appendicularia |  | *Oikopleura* spp. | KG1611 | KG1611 | KG1611, KG1711 |  | 3 | 3 | 6 |
| Thaliacea |  | Thaliacea spp. | NS1706, KG1711 | - | KG1711 |  | 7 | - | 1 |
|  |  | *Thalia democratica* | - | KG1611 | - |  | - | 3 | - |
| Chaetognatha |  | *Sagitta* spp. | NS1706, KG1611 | KG1711 | KG1611 |  | 5 | 3 | 3 |
| Hydrozoa |  | Hydrozoa spp. | KG1711, KG1906 | KG1511 | - |  | 8 | 2 | - |
|  |  | Muggiaea spiralis | - | KG1611 | - |  | - | 2 | - |
| Gastropoda |  | Gastropoda spp. | KG1711 | - | - |  | 7 | - | - |
|  |  | *Creseis acicula* | - | KG1611 | - |  | - | 2 | - |
|  |  | *Hyalocylis striata* | - | KG1711 | - |  | - | 1 | - |
| Polychaeta |  | Polychaeta spp. | NS1704, NS1706, KG1906 | KG1711 | - |  | 9 | 3 | - |

Supplementary Table S2. Information on locations, gears and environments for water and mesozooplankton samples. WT_200_: Mean temperature above 200 m. SAL_200_: Mean salinity above 200 m. CSW: Continental slope water. KR: Kuroshio path. OKR: Offshore of Kuroshio path. T-NORPAC: Twin-type North Pacific Standard net. S-NORPAC: Single-type North Pacific Standard net. -: No data.

| Station | Area | Location | | Date | Gear | | Layer | WT200 | SAL200 |
| --- | --- | --- | --- | --- | --- | --- | --- | --- | --- |
|  |  | Longitude | Latitude |  | Water | Mesozooplankton | （m） | （ºC） |  |
| SK4 | CSW | 30.54.058N | 130.22.631E | 170404 | - | T-NORPAC | 200 | 16.943 | 34.685 |
| SK4 | CSW | 30.54.001N | 130.22.565E | 170629 | - | T-NORPAC | 200 | 21.089 | 34.536 |
| SK4 | CSW | 30.53.980N | 130.22.453E | 171108 | - | T-NORPAC | 200 | 20.235 | 34.525 |
| A2 | CSW | 30.39.986N | 129.25.010E | 161108 | X-Niskin | T-NORPAC | 200 | 20.034 | 34.511 |
| A3 | CSW | 30.30.000N | 129.22.500E | 151116 | - | T-NORPAC | 200 | 21.292 | 34.694 |
| A5 | KR | 30.10.594N | 129.21.785E | 151116 | - | T-NORPAC | 200 | 22.993 | 34.844 |
| A5 | KR | 30.09.716N | 129.18.133E | 161106 | X-Niskin | T-NORPAC | 200 | 21.958 | 34.589 |
| A5b | KR | 30.08.747N | 129.17.251E | 161108 | - | T-NORPAC | 200 | 24.010 | 34.689 |
| A5 | KR | 30.10.001N | 129.17.522E | 171103 | - | T-NORPAC | 200 | 20.725 | 34.547 |
| A5 | KR | 30.10.033N | 129.17.573E | 171107 | - | T-NORPAC | 200 | 23.322 | 34.563 |
| A5' | KR | 30.10.855N | 129.17.248E | 171107 | - | T-NORPAC | 200 | 23.170 | 34.657 |
| A6 | KR | 30.00.204N | 129.15.316E | 161106 | - | T-NORPAC | 200 | 23.780 | 34.669 |
| A6b | KR | 30.00.011N | 129.15.350E | 161108 | - | T-NORPAC | 200 | 24.219 | 34.832 |
| A6 | KR | 29.59.976N | 129.15.186E | 171103 | - | T-NORPAC | 200 | 23.162 | 34.554 |
| A6 | KR | 30.00.009N | 129.14.963E | 171107 | - | T-NORPAC | 200 | 24.317 | 34.677 |
| A8 | OKR | 29.19.484N | 129.09.453E | 171106 | - | T-NORPAC | 200 | 24.400 | 34.734 |
| A8' | OKR | 29.26.317N | 129.12.442E | 171106 | - | T-NORPAC | 200 | 25.210 | 34.691 |
| A9 | OKR | 28.59.965N | 128.59.954E | 161107 | X-Niskin | T-NORPAC | 200 | 24.077 | 34.850 |
| A9b | OKR | 29.00.020N | 128.59.976E | 161107 | - | T-NORPAC | 200 | 23.812 | 34.802 |
| A9 | OKR | 29.00.041N | 128.59.936E | 171106 | - | T-NORPAC | 200 | 24.611 | 34.711 |
| TN05 | KR | 29.39.247N | 128.54.039E | 190620 | - | S-NORPAC | 20 | 28.030 | 34.530 |
| TN06 | KR | 29.54.039N | 127.52.922E | 190620 | - | S-NORPAC | 20 | 26.665 | 34.205 |

Supplementary Table S3. Contents and their amounts (µL) to be used for each PCR. -: No content.

| Contents | 1^st^ PCR | 2^nd^ PCR | 3^rd^ PCR |
| --- | --- | --- | --- |
| DW | 7.0 | 7.9 | 7.9 |
| X10 Buffer | 1.5 | 1.5 | 1.5 |
| dNTP | 1.5 | 1.5 | 1.5 |
| MgSO_4_ | 0.9 | 0.9 | 0.9 |
| KOD | 0.3 | 0.3 | 0.3 |
| F Primer (5 μM) | 0.9 | 0.45 | ― |
| R Primer (5 μM) | 0.9 | 0.45 | ― |
| F Primer (10 μM) | ― | ― | 0.45 |
| R Primer (10 μM) | ― | ― | 0.45 |
| Template (1 ng μl^-1^) | 2.0 | ― | ― |
| Template (1^st^ PCR product X20) | ― | 2.0 | ― |
| Template (2^nd^ PCR product X20) | ― | ― | 2.0 |

Supplementary Table S4. Temperature (ºC), duration and repeats of thermal cycler at each PCR step.

| Step | Temperature | Duration | Repeats |
| --- | --- | --- | --- |
|  | (ºC) |  |  |
| 1^st^ PCR | 94 | 2 min |  |
|  | 98 | 10 sec |  |
|  | 56 | 30 sec | 25 |
|  | 68 | 60 sec |  |
|  | 68 | 7 min |  |
|  | 4 | ∞ |  |
|  |  |  |  |
| 2^nd^ PCR | 94 | 2 min |  |
|  | 98 | 10 sec |  |
|  | 50 | 30 sec | 8 |
|  | 68 | 60 sec |  |
|  | 68 | 7 min |  |
|  | 4 | ∞ |  |
|  |  |  |  |
| 3^rd^ PCR | 94 | 2 min |  |
|  | 98 | 10 sec |  |
|  | 59 | 30 sec | 8 |
|  | 68 | 60 sec |  |
|  | 68 | 7 min |  |
|  | 4 | ∞ |  |


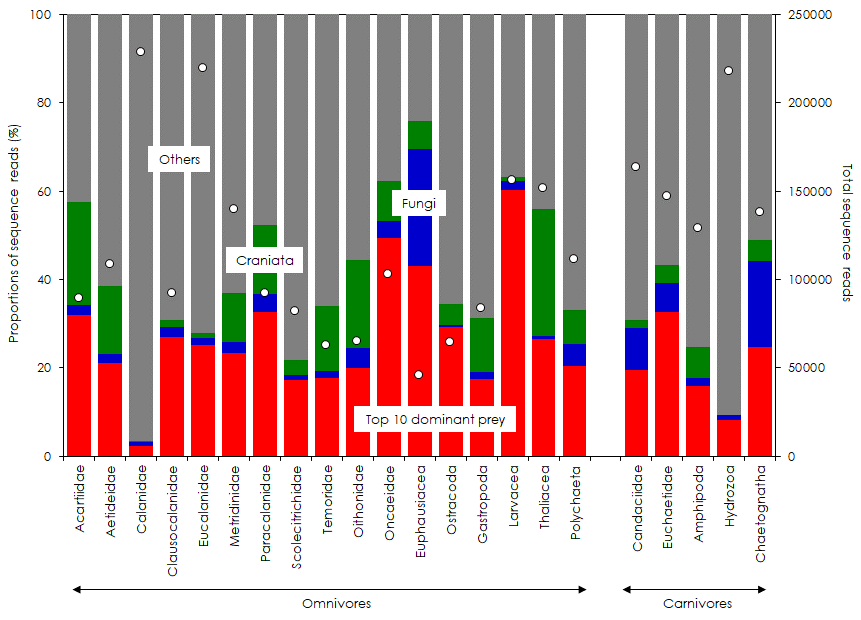


Supplementary Fig. S1. Total sequence reads (open circles) and their proportions of ten dominant prey (red), fungi (blue), craniata (green) and other OTUs (gray) for mesozooplankton gut-content DNA in the Kuroshio of the East China Sea and its neighboring waters. Note that total sequence reads and their proportions were averaged for gut-content DNA of five replicates at each taxonomic group.


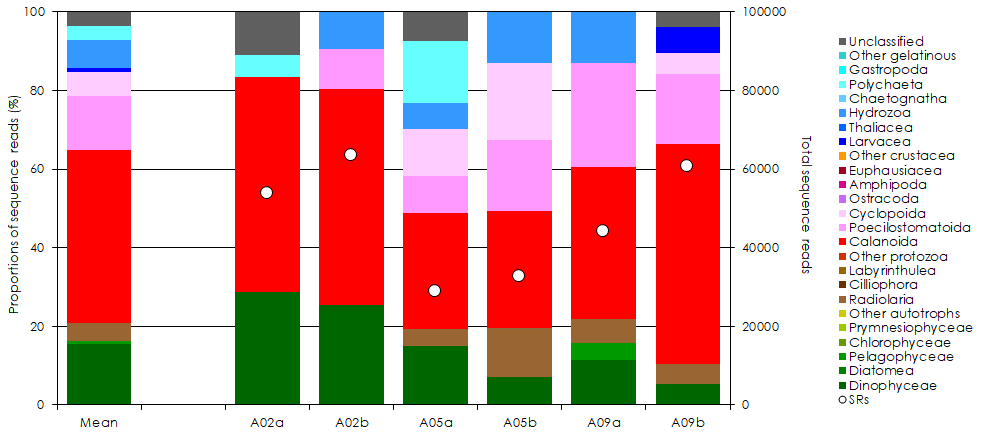


Supplementary Fig. S2. Total sequence reads (SRs: open circles) and their proportions of ten dominant prey OTUs (column) and in water sample in the Kuroshio of the East China Sea and its neighboring waters. Mean: average among the six samples.


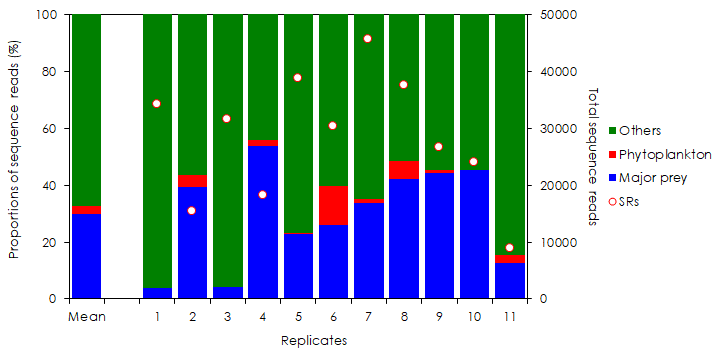


Supplementary Fig. S3. Total sequence reads (SRs: open circles) and their proportions of ten dominant prey OTUs (column) in gut content DNA of chaetognaths in the Kuroshio of the East China Sea and its neighboring waters. Mean: average among the eleven replicate samples.
